# Book Reviews

**Published:** 1913-06

**Authors:** 


### BOOK REVIEWS

---

U. S. PUBLIC HEALTH SERVICE. Annual Report 1912 (fiscal year ending June 30.)

There is probably no public medical service of the country which does the actual amount of work which this accomplishes. Over 50,000 patients are treated annually, nearly 15,000 being seriously sick or injured, so as to require hospital care. Over 10,000 physical examinations are also made. In addition, the investigation and management of epidemic diseases including infections following lines of commerce and transportation which have, happily, been prevented from becoming epidemic, represent a vast amount of work and a great saving of expense to the country. Furthermore, the Service carries on considerable research work and gathers statistics. This is accomplished with 138 commissioned officers and 227 acting assistant surgeons, the former having been increased by three and the latter reduced by 21 since the year before. Assistants of various kinds bring the total personnel of the Service to 1456.

---

SYSTEMATIC CASE-TAKING. Henry Lawrence McKisack, M. D., M. R. C. P., Belfast. Published by Paul B. Hoeber, 69 E. 59th St., N. Y.; 166 pages; $1.50.

History taking, general inspection, examination of regions and systems, and certain special considerations as of the Wassermann reaction, are discussed, always succinctly and briefly. Curiously enough, the author presents no elaborate system of blanks for records—which is a wise omission, as each physician can best establish his own system with reference to nature of practice and various personal and local factors.

---

SIGHT SAVING AS A NATIONAL MOVEMENT. REPORT OF THE COMMITTEE ON PREVENTION OF BLINDNESS of the Council on Health and Public Instruction of the A. M. A.

The first of these pamphlets is by Dr. F. Parke Lewis of Buffalo; the latter is the report of the committee of which he was chairman. When we consider that a great many normal individuals, given their choice between death and blindness, would prefer the former, the intense humanitarian interest of the noble work to prevent blindness, is apparent. The expense to the state is also an important factor. Much of the literature on this subject has reminded the reader of a harp with one string. No writer can neglect the terrible ravages of the gonococcus, but these pamphlets are refreshing in discussing a great variety of causes and methods of prevention of blindness.

---

THE EASTON SANITARIUM.

This is a copiously illustrated pamphlet, prepared under the direction of the Superintendent, Dr. C. Spencer Kinney of Easton, Pa. The views are so alluring that we regret having no time for a nervous breakdown.

---

ANNUAL REPORT OF THE AMERICAN TELEPHONE AND TELEGRAPH CO. for the year 1912.

The first exhibition of the telephone was at the Centennial Exhibition in 1876, where it attracted little attention. Even as late as 1890, there were only 200,000 telephones in use in the United States, the rapid initial growth in the 80's having been checked by the introduction of the a la carte method. In 1900

less than 500,000 stations were established. Following the expiration of the patents which prevented competition, and the institution of slot machines at a nickle instead of a dime for a message (the rate is a penny in England) with corresponding decrease in rentals and the return to the American plan of a flat rate in most cities, the Bell Company alone now has almost seven and a half million telephone stations, not to mention the enormous growth of independent services. The net revenue has increased from less than five and a half millions in 1900 to 32,000,000 in 1912. The average net profit per telephone station has been reduced from over $8.00 to about $4.50.

Few reports contain such valuable lessons in economics. Competition has paid the consumer, not to mention the capitalists' interests, both by the direct extension of service and by compelling good service and a reduction of rates. Speaking generally, it has paid the men who have borne the burden of a double telephone system, if they had never received or sent a message over the independent lines. It has paid them in actual net saving of expense, two companies having demonstrated that they could live cheaper than one even without the harmony of interests supposed by optimists to bring about this happy result in wedded life. It has paid them in the more rapid and more efficient service, not to mention wear and tear on the nervous system. One of our happiest recollections is of a Bell telephone magnate blaspheming at his own employees and service in the dark days of the beginning of the century when the expense of delivering a message by telephone meant the same cost as a double car fare and not much saving of time over the latter method. Now, thanks to competition, all this has been changed.

Strangely enough, competition has even benefitted the Bell interests; in spite of the pro rata reduction of net profits now amounting to only about 15% of an average rental, the aggregate net profit has increased over 600% as compared with 1900, while the aggregate capital invested has increased only about 250%.

But what an enormous saving in capital, rentals, and what an enormous saving of time, convenience, comfort and even life would have been effected if there had been some way of controlling the telephone business thirty years ago, as it has recently been controlled by competition and by legislation.

Perhaps we ought to add that this review does not consist entirely of extracts from the pamphlet itself.

---

THE DOCTOR'S DILEMMA, by George Bernard Shaw.

It may be remembered that this section of the JOURNAL formerly bore the broader title of "Books and Authors," and, while it is usually limited to reviews of new books, it is not necessarily so restricted. Indeed, some months ago we reviewed a book over a century old, and hope to have that pleasure again. The Doctor's Dilemma deals with the problem of selection of patients in the preliminary stage of a sure cure for tuberculosis, when there was a shortage of the remedy. It is peculiarly appropriate at this time when so much interest centers in Friedmann's claims. Curiously enough, the author omits altogether the consideration of the intensely dramatic and inevitable scepticism in the profession, and the resulting conflict of opinion. Shaw is usually regarded as hostile to the medical profession and he is certainly a severe critic in his tediously numerous prefaces and preambles, but, in the play itself, with due allowance for the almost necessary exaggeration of the stage, his portrayal of different types of doctors is founded on fact and is, in the main, friendly. The greatest interest seems to us to lie in his depiction of the ungrateful dead beat.

---

**The Surgical Clinics of John B. Murphy, M. D., at Mercy Hospital, Chicago. Volume II, number 1 (February, 1913). Octavo of 179 pages; illustrated. Philadelphia and London: W. B. Saunders Company, 1913. Published bi-monthly. Price per year, paper, $8.00; cloth, $12.00.**

This number opens with an address and operation by Mr. W. Arbuthnott Lane of London on the open treatment of Fractures. About half of the remaining clinics are devoted to Murphy's bone surgery. The rest include examples of a great variety of surgical lesions. This "case-system" in surgery is as interesting as in medicine, but possesses similar limitations as to comprehensiveness.

---

**A Privileged Medical Class. Dr. G. Frank Lydston, Chicago; reprint from the *Southern Practitioner*, February, 1913.**

Dr. Lydston has done valuable service as the Advocatus Diaboli of the profession. Sometimes his arguments are convincing, but we cannot share his foreboding that the Medical Reserve Corps of the U. S. A. is a scheme of the Medical Octopus. He states that the "first batch of appointments comprised the Editor-Manager-Boss of the A. M. A. and twelve of his official family," only one of whom had ever worn any uniform save the "collar" of the A. M. A., and that every local officer of the A. M. A. is now in the Chicago branch. He objects to the nominal nature of the examination and the exemption from state board examinations, in case of removal to another state. Otherwise expressed, this Corps is made up of prominent medical men, interested in professional matters; the Government recognizes their high professional standing, as a substitute for the formal examination necessary and proper in appointing young men to permanent salaried positions; and gives in return for their services, a privilege which very few are likely ever to use.

---

Private Duty Nursing. Katharine DeWitt, R. N., Assistant Editor of the *American Journal of Nursing;* J. B. Lippincott Co., Philadelphia; 244 pages, $1.50.

This book deals both with practical matters and considerations of ethics, deportment, business interests, etc. It contains much useful advice and is worthy of the attention, not only of nurses, but of physicians.

---

Epidemic Cerebro-Spinal Meningitis. Abraham Sophian, M. D., Kansas City; C. V. Mosby Publishing Co., St. Louis; 272 pages, illustrated; $3.00.

This is stated to be the only monograph in the English language on this disease. Dr. Sophian's experience in the N. Y. Research Laboratory has fitted him to discuss the subject as an authority. Every phase of the subject from the history of the disease as an epidemic, first recognized in 1805, to vaccine therapy and prophylaxis is treated, is given. A table of about 3,300 cases treated by various physicians with vaccine is given, showing an average mortality of about 30% as contrasted with a general average of about 70% for other methods.

---

Les Epanchements Du Pericarde (Pericardial Effusions). Dr. Germain Blechmann, Paris; J. B. Balliere et Fils, Paris; 350 pages, illustrated; paper covers; price not stated.

Beside a critical review of diagnostic points, including X-rays, clinical manifestations, therapy, pathology and sequellae, special attention is given to the method of Epigastric Puncture of Marfan. This is a valuable work and very easily read by those who have even a slight acquaintance with French.

---

Clinical Laboratory Methods. Roger S. Morris, A. B., M. D., St. Louis; D. Appleton & Co., N. Y.; 340 pages, illustrated; $3.00.

This is an excellent, condensed treatise, space being economized by sententious literary style rather than by omissions.

---

Transactions of the Fourteenth Annual Meeting of the Gastro-Enterological Association.

This is an assemblage of reprints in book form, with a list o members, constitution and by-laws. The meeting is the one at

Philadelphia in 1911. A considerable number of independent topics are covered and the matter is of the greatest value.

---

Proceedings of the Royal Academy of Medicine, Vol. 6, No. 5, March, 1913. Published by Longmans Green & Co., London; 7/6d.

This issue of the proceedings consists of a large paper-bound volume containing the general papers of a miscellaneous nature, arranged by sections, and a smaller supplement containing the first part of a symposium on Alimentary Toxaemia.

---

The Operating Room and the Patient (Third Edition Rewritten and Enlarged), by Russell S. Fowler, M. D., Chief Surgeon First Division, German Hospital, Brooklyn, New York. Octavo volume of 611 pages with 212 illustrations; Philadelphia and London; W. B. Saunders Company, 1913; cloth; $3.50 net.

While this book makes no pretensions to being a systematic treatise on surgery, the title suggests a much more modest field than is actually covered. The different kinds of operation are taken up in order and full directions for preparation of operating room and patient, for safeguarding the patient during and after operation, etc., are given in detail. Many hints as to diagnosis and choice of operation are also given and, indeed, there is scarcely any part of surgery on which light is not thrown.

---

Insurance Medicine. Being suggestions to Medical Examiners; Henry H. Schroeder, M. D., New York, Editor Insurance Department *Medical Record*. Wm. Wood & Co.; 150 pages; $2.00.

This book is a reprint of the articles contributed by Dr. Schroeder to the *Medical Record* and discusses various problems of interest to the insurance examiner—and there are few physicians who do not hold or do not hope to hold positions of this nature. The author's experience as Director of the Mutual Life Insurance Co. amply qualifies him for the work. In passing, it is pertinent to remark that of late years the influence of life insurance companies not only in securing a more scientific and better inforced system of vital statistics but in educating the profession and the public along hygienic and sanitary lines, has been a very important factor.

